# Editorial: Computational and experimental protein variant interpretation in the era of precision medicine

**DOI:** 10.3389/fmolb.2024.1363813

**Published:** 2024-01-15

**Authors:** Tiziana Sanavia, Paola Turina, Silvia Morante, Valerio Consalvi, Arthur M. Lesk, Constantina Bakolitsa, Daniele Dell'Orco

**Affiliations:** ^1^ Department of Medical Sciences, Computational Biomedicine Unit, University of Torino, Torino, Italy; ^2^ Department of Pharmacy and Biotechnology, University of Bologna, Bologna, Italy; ^3^ Department of Physics, University of Roma Tor Vergata, Roma, Italy; ^4^ Istituto Nazionale di Fisica Nucleare, University of Roma Tor Vergata, Roma, Italy; ^5^ Department of Biochemical Sciences “A. Rossi Fanelli”, Sapienza University of Roma, Roma, Italy; ^6^ Department of Biochemistry and Molecular Biology, The Pennsylvania State University, University Park, PA, United States; ^7^ Department of Plant and Microbial Biology and Center for Computational Biology, University of California, Berkeley, Berkeley, CA, United States; ^8^ Department of Neurosciences, Biomedicine and Movement Sciences, Section of Biological Chemistry, University of Verona, Verona, Italy

**Keywords:** single-point mutation, protein stability, genotype-phenotype correlation, molecular dynamics simulation, kinetics, pathogenicity, variant interpretation, machine learning

Most traits of the human phenotype depend on the combination of various genetic factors with environmental influences, and a major challenge is the understanding of the relationship among genetic and phenotype variations (Casadio et al., 2011). In the last years, both advancements in human genome sequencing technologies and the creation of databases collecting information on human variations at the gene and protein levels have hugely enhanced the investigations on the role of these variations in determining health and disease (Austin-Tse et al., 2022). At the same time, the increasing amount of data generated by these resources are requiring new accurate and reliable computer-aided tools to predict phenotype–genotype associations (Brandes et al., 2023; Cheng et al., 2023).

Efficient and powerful analytical methods are necessary for the discovery of unknown etiologies, which is important for rare diseases (Greene et al., 2023). Licata et al. highlighted the most relevant online resources and computational tools for single-nucleotide variant interpretation that can enhance the diagnosis, clinical management, and development of treatments for rare disorders.

A large number of computational methods have been developed for the identification of potentially pathogenic missense mutations. An example provided in this Research Topic is PON-All, a machine learning tool that exploits features of evolutionary conservation, changes in physicochemical properties of amino acids, and biological function annotations from Gene Ontology. The novelty, introduced by Yang et al. with this tool, was to improve the variant interpretation through the inclusion of non-human variants in the learning process, achieving high accuracy on a blind test set.

A complementary approach to understand the effects of missense mutations is by computational predictors of stability. Protein stability perturbations have already been associated with pathogenic missense variants, and they have been shown to significantly contribute to the loss of function in haploinsufficient genes (Birolo et al., 2021). The effects of these variants on protein stability can be measured as the difference in the free energy change of unfolding (ΔΔ*G*) between the mutated protein and its wild-type form. Predicting protein stability changes upon genetic variations is still an open challenge (Rollo et al., 2023). Current tools, which can either require the knowledge of the protein tertiary structure or rely on protein sequences only (Pancotti et al., 2021), are less accurate in predicting stabilizing variations than destabilizing ones (Pancotti et al., 2022). Benevenuta et al. investigated possible reasons for such a difference by focusing on the relationship between experimentally measured ΔΔ*G* and some protein properties (protein structural information, different physical properties, and statistical potentials). The results highlighted both the need to design predictive methods able to exploit input features highly correlated with the stabilizing variants and the importance of evaluating these tools on stabilizing, neutral, and destabilizing variants separately. Since this classification is associated with the sign of the protein melting temperature variation, Nobili et al. proposed a full atomistic protein description able to improve the estimation of free energy by modeling its change as a function of the number of hydrogen bonds computed using well-tempered metadynamics and maximal constrained entropy. The authors found a good agreement in the sign of representative values of ΔΔ*G* upon unfolding and the sign of the shift in the melting temperature compared to experimental results.

Comparing experimental characterization and computational predictions, Pacheco-Garcia et al. investigated naturally occurring variants of NAD(P)H:quinone oxidoreductase 1 (NQO1), a multifactorial protein associated with an increased risk of developing cancer and neurological disorders. The authors used computational tools to probe 5,187 variants, and the effects of the clinically relevant missense NQO1 variants were then experimentally characterized in terms of protein levels during bacterial expression, solubility, thermal stability, and coenzyme binding.

Disease-causing variants are supposed to directly affect experimentally measurable features, such as protein function and stability, and the kinetics and thermodynamics of protein–protein recognition, interaction, and binding. Morante et al. used circular dichroism, fluorescence spectroscopy, and melting temperature measurements to investigate key structural aspects of the interaction between wild-type frataxin and some of its variants found in cancer tissues upon Co2+ binding, highlighting the peculiar role of the N-terminal disordered tail in modulating the protein ability to interact with the metal. Dal Cortivo et al. provided a comprehensive biophysical investigation of calmodulin (CaM) by assessing structural, thermodynamic, and kinetic properties of protein–peptide interactions, involving two protein variants associated with congenital arrhythmia (N97I and Q135P) and a highly conserved CaM-binding region in ryanodine receptors RyR1 and RyR2. Specifically, the integration of spectroscopic investigation with molecular dynamics (MD) simulations and protein structure network analysis showed that these disease-associated CaM mutations alter CaM selectivity for the specific RyR channel.

The impact of MD simulations in molecular biology and drug discovery has expanded dramatically in recent years (Hollingsworth and Dror, 2018) since they capture the behavior of proteins and other biomolecules in full atomic detail and at a very fine temporal resolution. Shinwari et al. applied MD to characterize the structural and functional impacts of high-risk non-synonymous single-nucleotide polymorphisms on the TCIRG1 protein, causing congenital neutropenia and osteopetrosis. The analysis identified 15 variants that are likely to be highly deleterious, significantly destabilizing the wild-type protein structure and function. Hashimi et al. systematically investigated the dynamic properties involved in the double-stranded DNA (dsDNA) recognition by the EBNA1 protein, a key nuclear antigen of Epstein–Barr virus (EBV). Stability, flexibility, structural compactness, hydrogen bonding frequency, and binding affinity were altered by disrupting the native protein–DNA contacts, thereby decreasing the binding affinity. Their results revealed hotspot residues (arginine substitutions R521A and R522A), which are likely to become crucial in designing structure-based drugs against EBV infections.

In conclusion, this Research Topic has provided an overview of the current progress in both computational and experimental research and of their interplay in the annotation and interpretation of protein variants to detect pathogenic variations, analyzing their effects at the molecular level. These studies may help to predict the risk of developing specific diseases, the susceptibility to environmental factors, and the personal response to specific drugs.

## References

[B1] Austin-Tse C. A. Jobanputra V. Perry D. L. Bick D. Taft R. J. Venner E. (2022). Best practices for the interpretation and reporting of clinical whole genome sequencing. npj Genomic Med. 7, 27–13. 10.1038/s41525-022-00295-z PMC8993917 35395838

[B2] Birolo G. Benevenuta S. Fariselli P. Capriotti E. Giorgio E. Sanavia T. (2021). Protein stability perturbation contributes to the loss of function in haploinsufficient genes. Front. Mol. Biosci. 8, 620793. 10.3389/fmolb.2021.620793 33598480 PMC7882701

[B3] Brandes N. Goldman G. Wang C. H. Ye C. J. Ntranos V. (2023). Genome-wide prediction of disease variant effects with a deep protein language model. Nat. Genet. 55, 1512–1522. 10.1038/s41588-023-01465-0 37563329 PMC10484790

[B4] Casadio R. Vassura M. Tiwari S. Fariselli P. Luigi Martelli P. (2011). Correlating disease-related mutations to their effect on protein stability: a large-scale analysis of the human proteome. Hum. Mutat. 32, 1161–1170. 10.1002/humu.21555 21853506

[B5] Cheng J. Novati G. Pan J. Bycroft C. Žemgulytė A. Applebaum T. (2023). Accurate proteome-wide missense variant effect prediction with AlphaMissense. Science 381. eadg7492. 10.1126/science.adg7492 37733863

[B6] Greene D. Pirri D. Frudd K. Sackey E. Al-Owain M. Giese A. P. J. (2023). Genetic association analysis of 77,539 genomes reveals rare disease etiologies. Nat. Med. 29, 679–688. 10.1038/s41591-023-02211-z 36928819 PMC10033407

[B7] Hollingsworth S. A. Dror R. O. (2018). Molecular dynamics simulation for all. Neuron 99, 1129–1143. 10.1016/j.neuron.2018.08.011 30236283 PMC6209097

[B8] Pancotti C. Benevenuta S. Birolo G. Alberini V. Repetto V. Sanavia T. (2022). Predicting protein stability changes upon single-point mutation: a thorough comparison of the available tools on a new dataset. Brief. Bioinform. 23, bbab555. bbab555. 10.1093/bib/bbab555 35021190 PMC8921618

[B9] Pancotti C. Benevenuta S. Repetto V. Birolo G. Capriotti E. Sanavia T. (2021). A deep-learning sequence-based method to predict protein stability changes upon genetic variations. Genes 12, 911. 10.3390/genes12060911 34204764 PMC8231498

[B10] Rollo C. Pancotti C. Birolo G. Rossi I. Sanavia T. Fariselli P. (2023). Influence of model structures on predictors of protein stability changes from single-point mutations. Genes 14, 2228. 10.3390/genes14122228 38137050 PMC10742815

